# Distinct Changes Occur in the Human Breast Milk Microbiome Between Early and Established Lactation in Breastfeeding Guatemalan Mothers

**DOI:** 10.3389/fmicb.2021.557180

**Published:** 2021-02-12

**Authors:** Emmanuel Gonzalez, Nicholas J. B. Brereton, Chen Li, Lilian Lopez Leyva, Noel W. Solomons, Luis B. Agellon, Marilyn E. Scott, Kristine G. Koski

**Affiliations:** ^1^Canadian Centre for Computational Genomics (C3G), Department of Human Genetics, McGill University, Montréal, QC, Canada; ^2^Microbiome Research Platform, McGill Interdisciplinary Initiative in Infection and Immunity (MI4), Genome Centre, McGill University, Montréal, QC, Canada; ^3^Institut de Recherche en Biologie Végétale, Université de Montréal, Montréal, QC, Canada; ^4^School of Human Nutrition, McGill University, Ste-Anne de Bellevue, QC, Canada; ^5^Center for Studies of Sensory Impairment, Aging and Metabolism (CeSSIAM), Guatemala City, Guatemala; ^6^Institute of Parasitology, McGill University, Ste-Anne de Bellevue, QC, Canada

**Keywords:** 16S rRNA gene, human breast milk, microbiome, lactation stage, metagenomics 16S

## Abstract

Human breast milk contains a diverse community of bacteria, but as breast milk microbiome studies have largely focused on mothers from high income countries where few women breastfeed to 6 months, the temporal changes in the breast milk microbiome that occur during later lactation stages have not been explored. For this cross-sectional study, microbiota from breast milk samples of *Mam*-Mayan mothers living in eight remote rural communities in the Western Highlands of Guatemala were analyzed. All mothers delivered vaginally and breastfed their infants for 6 months. Breast milk from 76 unrelated mothers was used to compare two lactation stages, either “early” (6–46 days post-partum, *n* = 33) or “late” (109–184 days post-partum, *n* = 43). Breast milk microbial communities were assessed using 16S ribosomal RNA gene sequencing and lactation stages were compared using DESeq2 differential abundance analysis. A total of 1,505 OTUs were identified, including 287 which could be annotated as putative species. Among several maternal factors, lactation stage explained microbiome variance and inertia in ordination with the most significance (*p* < 0.001). Differential abundance analysis identified 137 OTUs as significantly higher in either early or late lactation. These included a general shift from *Staphylococcus* and *Streptococcus* species in early lactation to *Sphingobium* and *Pseudomonas* species in late lactation. Species enriched in early lactation included putative commensal bacteria known to colonize the infant oral and intestinal tracts whereas species enriched in late lactation had a uniform functional trait associated with aromatic compound degradation. Differentially abundant species also included several species which have not previously been reported within breast milk, such as *Janthinobacterium agaricidamnosum*, *Novosphingobium clariflavum*, *Ottowia beijingensis*, and *Flavobacterium cucumis.* These discoveries describe temporal changes to the breast milk microbiome of healthy Guatemalan mothers from early to late lactation. Collectively, these findings illustrate how studying under-represented human populations might advance our understanding of factors that modulate the human milk microbiome in low and middle income countries (LMIC).

## Introduction

Human breast milk is a source of macronutrients and micronutrients essential for infant nutrition (Cunningham et al., 1991; Walker, 2010; Andreas et al., 2015). Research has established that breast milk also provides infants with a continuous source of commensal and potentially beneficial bacteria which can act to inoculate the infant respiratory and gastrointestinal tracts (Fernández et al., 2013; McGuire and McGuire, 2017). Predominant genera commonly reported include *Staphylococcus*, *Streptococcus, Pseudomonas, Cutibacterium* (formally *Propionibacterium*), or *Bifidobacterium* (Jeurink et al., 2012; Fitzstevens et al., 2017). Recently, metagenomic sequencing studies have begun to characterize the diverse bacterial species in breast milk to better understand how this process might be associated with infant and long-term health (Biagi et al., 2017; Ruiz et al., 2019; Sakwinska and Bosco, 2019).

Several maternal factors are thought to modify bacterial communities in human breast milk. One study reported differences in bacteria (observed at the genera level) related to stage of lactation, maternal BMI and mode of delivery (Cabrera-Rubio et al., 2012), but other studies have reported little or no differences in relative abundance of genera due to maternal age, parity, mode of delivery, or infant sex (Urbaniak et al., 2016; Williams et al., 2017). Given the advancements in resolution of microbial barcoding technologies (Knight et al., 2018; Bolyen et al., 2019; Gonzalez et al., 2019), coupled with the need to identify the factors that influence breast milk microbiota (McGuire and McGuire, 2017), the purpose of the study was to determine if human milk microbiota differed by maternal age, BMI, parity, breastfeeding practices and lactation stage. Breast milk samples were collected from unrelated lactating mothers living in the remote Western Highlands of Guatemala during early (6–46 days postpartum) or established lactation (109–184 days postpartum) as previously described (Wren et al., 2015).

## Materials and Methods

### Study Site and Participants

This cross-sectional study was part of collaboration between McGill University and the Center for Studies of Sensory Impairment, Aging and Metabolism (CeSSIAM) in the Republic of Guatemala. Field studies were conducted in eight rural Mam-speaking communities of the San Juan Ostuncalco region in Guatemala (Chomat et al., 2015) between June 2012 and January 2013. Lactating mothers were identified and invited to participate by community health workers. Individuals who had been treated with antibiotics were excluded. All mothers delivered vaginally and breastfed their infants. Samples from participating mothers with infants aged from 6 to 46 (*n* = 33) and 109–184 (*n* = 43) days postpartum were used to compare the milk microbiome during early and late lactation. Breastfeeding practices were also compared. Exclusively breastfeeding was defined as infants receiving only breast milk; predominant breastfeeding was defined as infants who breastfed but also received water, most commonly as “agüitas” (*herbal teas)*, and mixed breastfeeding was defined as infants who breastfed but were also given solid foods in addition to aguitas (Wren et al., 2015).

### Breast Milk Sample Collection

Prior to sample collection, the nipple and areola of the breast were cleaned with 70% ethanol. Breast milk samples were collected during a 3 h time window in the morning from the breast (not recently used for breastfeeding) via full manual expression by a trained midwife. Milk was collected into sterile 60 ml plastic vials and immediately stored on ice. Samples were partitioned into 15 ml tubes at the field laboratory (−30°C) prior to transfer on dry ice to McGill University where they were stored at −80°C (Li et al., 2016).

### 16S rRNA Gene Amplification, Sequencing, and Bioinformatics

DNA extraction was performed using 1ml of milk with DNeasy Blood and Tissue mini kit from Qiagen according to the manufacturer’s protocol by Genomic Quebec Innovation Centre. For PCR, a target region of ∼526 bp based on the *Escherichia coli* 16S rRNA gene covering the V1–V3 was amplified with the primers 27F (3′-AGAGTTTGATCCTGGCTCAG-5′-TTACCGCGGCTGCTGGCAC-5′) (Cabrera-Rubio et al., 2012; Mediano et al., 2017; Lackey et al., 2019). Amplification used the following conditions: initial denaturation 94°C for 2 min, denaturation 94°C for 30 s, annealing 58°C for 30 s, extension 72°C for 30 s, final extension 72°C for 7 min, 4°C hold, over 35 cycles. Amplification was conducted by the Genomic Quebec Innovation Centre at McGill University and sequencing was performed using the Illumina MiSeq platform. Reagent controls were below the detection limit used by Genomic Quebec Innovation Centre for quality assurance. The Anchor pipeline (Gonzalez et al., 2019) was used to process amplicon sequences. Briefly, Mothur (Schloss et al., 2009) was used to align and dereplicate sequences before high count OTU selection at a count threshold of 36 across all samples. NCBI 16S rRNA RefSeq, NCBI non-redundant nucleotide, SILVA, and the Ribosomal Database Project (RDP) databases were used to annotate OTUs using BLASTn with criteria of >99% for identity and coverage. When a BLASTn return had 100% identity and coverage hits across multiple database, priority was given to NCBI 16S rRNA RefSeq due to the high standard of curation. Low counting amplicons (<36 counts) were binned to high-count OTUs at a lower threshold of >98% identity/coverage. Multiple, equally good (highest identity/coverage), annotation was retained and reported. All annotation, and in particular species calls, should be considered putative even when sharing 100% sequence identity to a single species due to database errors.

Alpha diversity of breast milk samples was measured using Shannon and inverse Simpson indices within Phyloseq R package (McMurdie and Holmes, 2013). Beta diversity was estimated based on variation stabilization normalized counts (rlog) using Bray-Curtis dissimilarity and the Constrained Analysis of Principal Coordinates (CAP) ordination method. Dispersion ellipses were drawn using veganCovEllipse function from Vegan package in R (Oksanen et al., 2017). Significant distance was evaluated between the groups using non-parametric analysis of similarities (ANOSIM) on normalized counts based on Bray distances (Vegan package). To characterize differentially abundant taxonomic units between groups of samples, parametric models developed in transcriptomics perform well when applied to microbiome biomarker data (uneven library sizes, sparsity, and sample representativity) (Jonsson et al., 2016; Weiss et al., 2017; Brereton et al., 2019; Gonzalez et al., 2019; Minerbi et al., 2019). DESeq2 procedure (Love et al., 2014a) was used to calculate differentially abundant taxonomic units. Taxonomic units tested with a false discovery rate (FDR or the expected proportion of false positive findings) <0.1 were considered significant (Anders et al., 2013; Love et al., 2014b, 2015; Gonzalez et al., 2019). Pearson’s correlation was calculated between rlog normalized OTU counts. Correlation matrices were sorted based on hierarchical clustering of OTUs and represented using R package corrplot (Wei and Simko, 2017).

## Results

### Characterization of Guatemalan Mothers

Participating mothers were randomly selected from 8 remote *Mam*-Mayan villages in the Western Highlands of Guatemala: Los Romero (28%), Buena Vista (25%), Los Alnonzo (13%), and <10% from each of Espumpujá, Los Lopez, Los Perez, La Unión, and La Esperanza. Population characteristics by lactation stage are summarized in Table 1. With regards to breastfeeding practices, 86% exclusively or predominantly breastfed; mixed feeding was more common in late lactation, increasing from 3 to 23.3%; regardless of breastfeeding practice, frequency per 24 h averaged 11 feeds per day, which aligns with current recommendations.

**TABLE 1:** Characteristics of Guatemalan mothers at each lactation stage.

	Lactation stages		
	
	Total mean ± *SD* or % (95% CI)	Early mean ± *SD* or % (95% CI)	Late mean ± *SD* or % (95% CI)
**Region (%)**			
Los Alonzo	13.1	18.2	9.3
Buena Vista	25.0	12.1	34.9
Espumpujá	2.6	6.1	0.0
Los López	3.9	9.1	0.0
Los Pérez	9.2	3.0	14.0
Los Romero	27.6	36.4	20.9
La Unión	9.2	6.1	11.6
La Esperanza	9.2	9.1	9.3
**Maternal age (years)**	23.2 ± 5.8	22.7 ± 5.5	23.6 ± 6.0
**Maternal height (cm)**	146.7 ± 5.1	146.2 ± 4.7	147.2 ± 5.4
**Maternal weight (kg)**	50.8 ± 7.6	50.2 ± 6.7	51.2 ± 8.3
**Maternal BMI (kg/m^2^)**	23.5 ± 3.1	23.5 ± 2.8	23.5 ± 3.4
**Parity**			
Primiparous	49.0	45.0	52.0
Multiparous	51.0	55.0	48.0
**Marital status (%)**			
Married	25.0	27.0	22.5
United	73.0	73.0	72.5
Single	3.0	0.0	5.0
**Education (%)**			
No	81.0	85.0	76.0
Primary education or higher	19.0	15.0	24.0
**Delivery location and attendant**			
Informal sector and midwife	78.0	85.0	73.0
Hospital and formal	22.0	15.0	28.0
**Infant feeding practices**			
Exclusive	47.4	51.5	44.2
Predominant	38.2	45.5	32.6
Mixed	14.5	3.0	23.3
Breastfeeding First hour	61.0	53.0	68.0
Frequency/24 h	11.9 ± 4.3	11.7 ± 4.7	12.1 ± 4
**Infant first food**			
Breast	88.0	88.0	87.5
Agüitas	11.0	12.0	10.0
Beverages	1.0	0.0	2.5

### Breast Milk Microbiome Community Differs Between Lactation Stages

Sample extractions yielded significantly different concentrations of total genomic DNA between early and late groups at an average of 11.9 ng μl (±3.2) compared to 3.0 ng μl (±0.6), respectively. A total of 1,505 OTUs were assembled and captured 6,294,781 sequence reads across all 76 breast milk samples. These could be annotated as 287 species (71% of reads), 180 genera and 23 family or higher taxa as well as 1,015 which couldn’t be recognized as 99% similar (in both identity and coverage) to any known taxa (termed TrueUnknowns). Of the 287 OTUs annotated as putative species, the average BLASTn return identity was 99.8% including 167 perfect hits (100% identity). At a phyla level, bacteria from Firmicutes, Proteobacteria, Actinobacteria, and Bacteroidetes were most prevalent in comprising 48, 44, 6, and 1% of sequenced amplicons, respectively (Supplementary File 1). The most abundant species across all samples were *Streptococcus salivarius* and *Novosphingobium clariflavum* making up 7.54 and 6.22% amplicons, respectively.

**FIGURE 1:**
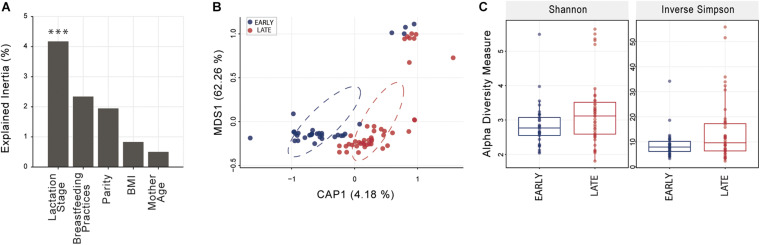
Breast milk community ordination and alpha diversity comparing early and late lactation microbiome. **(A)** Variation explained by the tested variables in constrained ordination (CAP). Significance testing using Monte Carlo permutation test (10,000 permutation; ***p < 0.001). **(B)** Lactation stage CAP, circles represent sample density relative to each group. **(C)** Alpha diversity indices were not significantly different (*t*-test, *p* > 0.05) between early (*n* = 33) and late (*n* = 43) lactation groups. See Supplementary File 1 for an overview of OTU taxonomy.

Distance-based ordination using sparsity filtered raw count matrix was performed with five different categorical variables; four were dichotomous (lactation stage, parity, BMI, and mother age) and one (breastfeeding practice) was based on three factors (exclusive, predominant, or mixed). Constrained Analysis of Principal Coordinates (CAP) analysis best segregated mothers based on lactation stage (Figure 1) and identified significant differences with Monte Carlo permutation testing between sample groups (10,000 permutations) at *p* < 0.001; parity, BMI, mother age and breastfeeding practice were not significant (*p* > 0.1 in all cases). Shannon and Inverse Simpson indices, used to estimate microbiome diversity, were not significantly (*t*-test *p* < 0.05) higher in late compared to early lactation stage breast milk (Figure 1).

**FIGURE 2:**
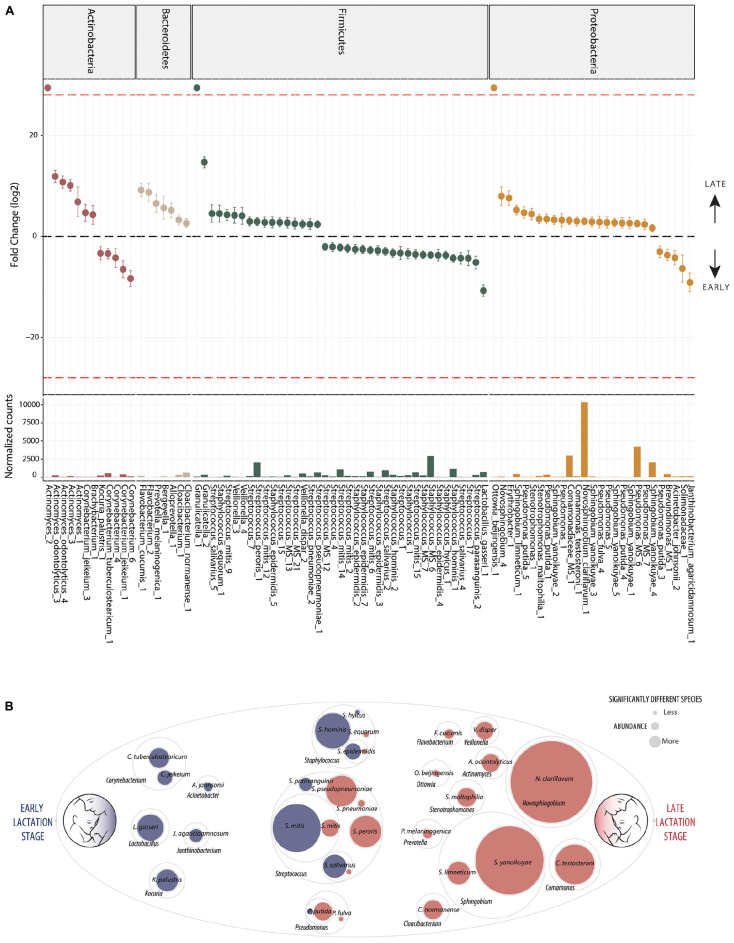
Differentially abundant bacteria associated to lactation stage. Differentially abundant OTUs between the early (*n* = 33) and late (*n* = 43) groups. **(A)** Fold change (FC log_2_) in relative abundance of significantly different (DESeq2) OTUs between groups and their normalized mean counts. Species are grouped by phylum. The dashed red line indicates “infinite” fold change, where an OTU had detectable counts in samples from only a single group. **(B)** An illustration of species-level differentially abundant bacteria between milk of early (blue) and late (red) lactation stages. Raw abundance is represented. The complete OTU table including relative abundance, annotation, count distribution, blast statistics, alternative database hits, and sequences are provided in Supplementary File 1.

**FIGURE 3:**
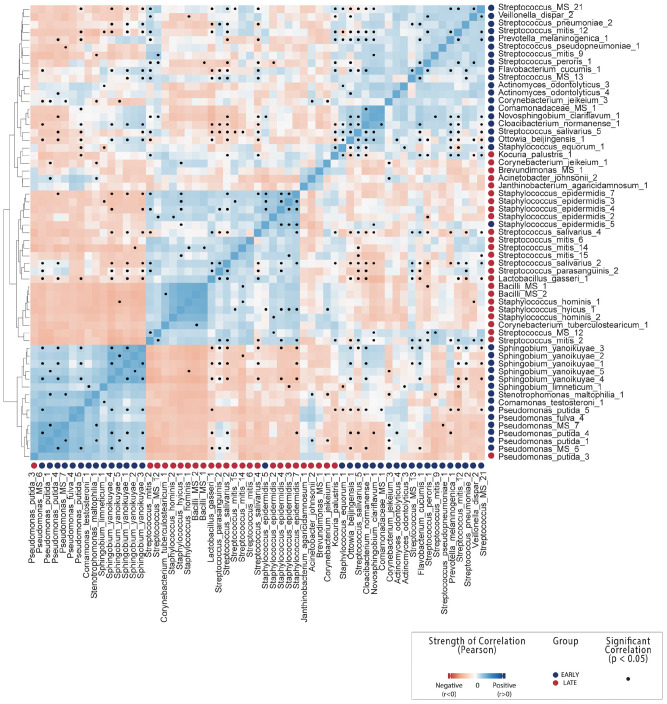
OTU-OTU correlations across all breast milk samples. Count correlation of all OTUs differentially abundant between early and late lactation stages which could be annotated at species level. Colors indicate positive (blue) or negative (red) correlation and black dots indicate significance (*p* < 0.05). See Supplementary File 1 correlations including non-differentially abundant of OTUs from mothers.

In contrast, differential abundance analysis using DESeq2 identified taxa which were significantly different in relative abundance between early and late lactation stages. In total, 137 OTUs were identified as significantly differentially abundant, including 39 which were in higher relative abundance in early stage milk and the remaining 98 higher in late stage milk (Figure 2 and Supplementary File 2). These could be annotated at various taxonomic levels, including: 60 species, 25 genera, 1 family as well as 52 which could not be recognized (TrueUnknowns). A general shift in differential abundance was observed with lower relative abundance in some taxa from Firmicutes in early stage milk which were replaced by taxa from Proteobacteria in late stage milk. This was driven by *Streptococcus* and *Staphylococcus* species present at higher relative abundance within early milk whereas *Sphingomonadaceae* and *Pseudomonas* were higher in late milk, with the majority of differentially abundant species in each respective taxa group being inversely correlated (Figure 3 and Supplementary File 1).

### Bacterial Species Associated With Early Lactation

Ten distinct *Staphylococcus* OTUs were identified as having significantly higher relative abundance in early milk, including the species: *S. hominis, S. epidermidis*, and *S. hyicus* (Figure 2). The most abundant *Staphylococcus* OTU (Staphylococcus_MS_6) was ambiguous and could represent one or more of the species *S. epidermidis*, *S. haemolyticus*, or *S. hominis*.

Seventeen differentially abundant OTUs with higher abundance in early lactation stage breast milk were identified as putative species within the genus *Streptococcus*. These included *S. mitis*, *S. parasanguinis*, *S. peroris*, *S. pneumoniae*, *S. pseudopneumoniae*, and *S. salivarius* as well as four OTUs which were ambiguous and could represent a number different of species. Beyond *Staphylococcus* and *Streptococcus*, other OTUs representing species identified as significantly higher in relative abundance in early milk included: *Corynebacterium tuberculostearicum, Corynebacterium jeikeiu, Lactobacillus gasseri, Acinetobacter johnsonii, Kocuria palustris*, and *Janthinobacterium agaricidamnosum*.

### Bacterial Species Associated With Late Lactation

The general pattern of higher *Staphylococcus* and specific *Streptococcus* species in early milk was also reflected by corresponding significant (*p* < 0.05) inverse correlations with *Sphingobium yanoikuyae*, *Pseudomonas putida*, *Stenotrophomonas maltophilia, Ottowia beijingensis*, and *Comamonas testosteroni*, all of which were significantly differentially abundant with higher relative abundance in late breast milk.

Five distinct *S. yanoikuyae* OTUs and a *S. limneticum* OTU were identified as significantly higher in late milk. The two most abundant OTUs, Sphingobium yanoikuyae_4 and Sphingobium yanoikuyae_5, likely represent distinct strains in sharing 100% identity with *Sphingobium yanoikuyae* S72 and ATCC 51230, respectively, with each strain harboring four identical gene copies. Additionally, a single *Novosphingobium clariflavum* OTU was identified as differentially abundant and higher in late breast milk. The *Novosphingobium clariflavum* OTU was particularly high in abundance in the majority of late breast milk samples and was the most abundant OTU in 51% of late milk samples, representing an average of 25% of raw reads.

Nine *Pseudomonas* OTUs were differentially expressed with eight having higher relative abundance in late milk. These included four *P. putida* and one *P. fulva* OTU as well as three ambiguous OTUs which could be *P. putida*, *P. fulva*, *P. parafulva*, and/or *P. gingeri*. *Stenotrophomonas maltophilia* (closely related to *Pseudomonas*), *Cloacibacterium normanense*, *Flavobacterium cucumis, Ottowia beijingensis* (absent from early milk samples), *Prevotella melaninogenica*, *Veillonella dispar, Actinomyces odontolyticus* as well as the highly abundant *Comamonas testosteroni*, were also each identified as significantly more abundant in late lactation stage breast milk.

A number of poorly characterized OTUs were also identified as having higher relative abundance in late milk. These were dominated by unknown sequences previously observed in samples from oral or saliva microbiome studies, such as: TrueUnknown_ 944 and TrueUnknown_ 978 (97.5% similar *Streptococcus sanguinis* SK1), TrueUnknown_967 and TrueUnknown_980 (98% similar *Prevotella* sp.), TrueUnknown_915 (98% similar *Granulicatella elegans*), as well as the high relative abundance TrueUnknown_926 which was 97% similar to a (challenging to culture) TM7 group bacterium often found in oral samples.

## Discussion

### General Characteristics

Breast milk supplies a continuous source of bacteria during lactation (Fitzstevens et al., 2017) and there is consensus that this provision inoculates the gastrointestinal tract with commensal or beneficial bacteria (Martín et al., 2004; Ramsay et al., 2004; Moossavi et al., 2019; Williams et al., 2019). However, this milk bacterial community is thought to be influenced by the communities present in other maternal tissues (e.g., skin and the gastrointestinal tract) as well as the infant oral cavity (e.g., “retrograde flow”) and the environment (Jost et al., 2014; LaTuga et al., 2014; Rodríguez, 2014). Despite this, the microbiome of breast milk is still poorly understood, and no consensus has been reached on the “core” genera microbiome (Jeurink et al., 2012; Fitzstevens et al., 2017) or if dynamic changes in this bacterial community might occur throughout lactation. In our study, unrelated mothers living in eight remote but distinct communities with little possibility of exchanging microbes among one another were studied. Moreover, this indigenous Guatemalan population was chosen because nearly 100% exclusively or predominantly breastfed for 6 months (Wren et al., 2015), complying with WHO recommendations to actively breastfeed during this period (World Health Organization, 2014). This is considerably higher than the global average of 41% of mothers breastfeeding for 6 months, which falls to as low as 26% in North America (UNICEF, 2018). Importantly, this study population also represented a cohort of mothers who are not from a high-income country, which currently dominates the research field. Thus, this study contributes toward identifying common characteristics and possible biases generated from a shortfall in participant diversity from under-represented communities in breast milk microbiome research.

### Early Lactation Stage Breast Milk Is Dominated by Commensal Bacterial Species

Although previous studies have reported differences in the human milk microbiome related to maternal BMI and stage of lactation (Cabrera-Rubio et al., 2012; Williams et al., 2017, 2019; Moossavi et al., 2019), few have characterized the milk microbiome by identifying changes in the relative abundance of putative species. The benefit of high-resolution microbiome analysis at species level is that, although many species remain poorly characterized, the potential for association to established functions is improved when compared to annotation at phyla, family or genera level. Species-level annotation however, even when sequences share 100% 16S rRNA gene fragment similarity to a single species, should be considered as putative as substantial mistakes exist in major repositories (Gonzalez et al., 2019). The two most abundant species identified in higher relative abundance in early lactation stage were the coagulase negative *Staphylococcus hominis* and *Staphylococcus epidermidis* (Figure 2), both of which are ubiquitous in humans and have been previously associated with opportunistic pathogenicity (Coates et al., 2014). The higher abundance of *S. hominis* and *S. epidermidis* in early milk is consistent with findings that non-virulent strains of these species are likely early gut pioneers of term infants, and may have the potential to reduce colonization by more virulent species (Soeorg et al., 2017). In our study, it was also interesting to note that although both species are recognized as common skin bacteria and common inhabitants of healthy breast milk environment (Heikkilä and Saris, 2003; Martín et al., 2007), their relative abundance was lower in late milk.

*Streptococcus mitis* and *Streptococcus salivarius* strains are usually considered commensal, are commonly isolated from milk (Kilian et al., 2014; Delorme et al., 2015; Sørensen et al., 2016) and have been identified as infant oral microbiome inhabitants within the first days after birth (Pearce et al., 1995). The OTUs from both species were generally in higher relative abundance in early milk (Figure 2). An exception to this was in *S. mitis*, where two of the six differentially abundant *S. mitis* OTUs (Streptococcus_mitis_9 and Streptococcus_mitis_12) had higher relative abundance in late milk. Interestingly, the most abundant *S*. *mitis* OTU in early (Streptococcus_mitis_14) and late (Streptococcus_mitis_9) lactation stages share 100% identity to distinct strains, *Strep mitis* RH_12363_08 and *Strep mitis* SK637, respectively. These OTUs, alongside the others identified as significantly varying between early and late lactation stages are most similar to different *S. mitis* phylogenetic sub-clades (Supplementary File 1; NCBI Genome Tree report), in line with previously described strain phylogeny (Rasmussen et al., 2016). Although little is known about functional variation between these strains, these results suggest that such variation does exist.

Species from the genus *Corynebacterium* are very commonly isolated from human breast milk and are generally considered commensal in nature (Hunt et al., 2011; McGuire and McGuire, 2017). The two species identified as higher in relative abundance in early milk could be a concern as both *C. tuberculostearicum* (which was first identified as leprosy-derived *Corynebacterium* and *C. jeikeium)* have historically been considered pathogenic (Riegel et al., 1994; Paviour et al., 2002; Hinić et al., 2012), although recent evidence has indicated that these species can be commensal (Damaceno et al., 2017). Similarly, *Acinetobacter johnsonii*, which has been previously, although not commonly, detected in breast milk and is known to utilize lactate (Bouvet and Grimont, 1986; Delgado et al., 2008), could be a potential health concern as it has been associated with mastitis (Patel et al., 2017). *Kocuria palustris*, first isolated from rhizosphere samples (Kovács et al., 1999), was also unexpected within early breast milk as the species has not previously been reported in breast milk samples; although the genera has been detected in both ruminant and human milk (Jeurink et al., 2012; Li et al., 2017; Lackey et al., 2019).

A number of species characterized as potentially beneficial to infant health were also identified as significantly enriched in early breast milk. *Lactobacillus gasseri* is well characterized as a species with some strains that produce bacteriocins (gassericin A) (Pandey et al., 2013; Mousa et al., 2017) and is widely considered a putative early colonizer of infant guts. It is subsequently heavily marketed as a probiotic due to the potential to provide host resistance to infections from pathogens such as *Listeria monocytogenes* (Kawai et al., 2004; Selle and Klaenhammer, 2013), although evidence of this function *in situ* has not been yet been generated. *L. gasseri* was significantly positively correlated with *S. salivarius* and *S. parasanguinis* here (Figure 3 and Supplementary File 1) and inversely correlated with the *Sphingobium* and *Pseudomonas* species in late milk microbiome. Another species with potential antimicrobial properties in significantly higher relative abundance in early lactation stage breast milk was *Janthinobacterium agaricidamnosum*, which produces an anti-fungal compound (jagaricin) with strong antifungal activity against major human fungal pathogens (Lincoln et al., 1999; Graupner et al., 2012, 2015). Although the species could potentially improve infant biotic resistance, it has not previously been observed in milk and is largely uncharacterized (jagaricin also has hemolytic activity; Fischer et al., 2019).

### Species Associated With Late Lactation Stage Breast Milk Have a Common Function

An inverse correlation between bacteria within the phyla Firmicutes and Proteobacteria has recently been observed in human breast milk (Moossavi et al., 2019). The major genera identified here as representing this shift toward Proteobacteria in later lactation (Figure 2), *Sphingobium* and *Novosphingobium*, are often found in human breast milk and have been previously identified as highly abundant together with *Pseudomonas* in milk of healthy mothers in a non-marker based metagenomic study (Jiménez et al., 2015). *Sphingobium yanoikuyae*, identified here as significantly higher in relative abundance in late breast milk, is a well-known soil bioremediation species with some strains having polycyclic aromatic hydrocarbons (PAH) degradation activity in the environment (Cunliffe and Kertesz, 2006; Kou et al., 2018) but was first isolated from human clinical samples (Yabuuchi et al., 1990). Recruitment of bacteria with such functionality could be a response to accumulation of xenobiotics such as PAHs and polychlorinated biphenyls, which can accumulate in fatty tissue and breast milk (Dekoning and Karmaus, 2000), although this would not explain association with lactation stage. On the other hand, this species has also been previously observed as associated with nipple aspirate fluid of healthy women in a breast cancer study, where the authors speculated that the PAH degrading activity may provide protection against cancer tumor development (Chan et al., 2016; Parida and Sharma, 2019). Finally, although *N. clariflavum* was very widely distributed throughout samples, the species has yet to be characterized in detail (Zhang et al., 2017) and has never been previously identified in breast milk samples. However, *Novosphingobium* have been isolated from a very wide range of habitats including soils, plants and water, and are known as aromatic hydrocarbon compound degraders expected within soils and rhizospheres contaminated with persistent organic pollutants (Kumar et al., 2017; Wang et al., 2018).

The pattern of hydrocarbon degradation functionality was consistent with the majority of species identified as significantly enriched in late breast milk (Figure 2). *Pseudomonas putida* strains are common soil and rhizosphere degraders of persistent organic pollutants which are used for bioremediation as it can metabolize aromatic hydrocarbons as a sole carbon source (Feist and Hegeman, 1969; Dunn and Gunsalus, 1973; Marqués and Ramos, 1993), including caffeine (Summers et al., 2015). While *Pseudomonas* species are commonly observed in metagenomic studies of healthy mothers (Jeurink et al., 2012; Fitzstevens et al., 2017), they are generally considered environmental contamination in human breast milk (Jiménez et al., 2015). The closely related *Stenotrophomonas maltophilia*, which is considered non-lactose fermenting, has previously been identified as an opportunistic pathogen associated with cystic fibrosis, where it can form mixed species biofilm with *Pseudomonas aeruginosa* (Esposito et al., 2017). Given an established capability to cooperatively associate with *P. aeruginosa*, it is interesting to note that the species was significantly correlated in our study with *P. putida* (Figure 3).

Consistent with the differential abundance of *Sphingobium yanoikuyae* and *P. putida*, strains of *Stenotrophomonas maltophilia* also have extensive hydrocarbon degradation activity (Singh and Fulekar, 2010; Arulazhagan et al., 2017). The same is true of *Cloacibacterium normanense*, which is often isolated from wastewater and can often grow on oily sludge as its sole carbon source (Cerqueira et al., 2012), and *Comamonas testosteroni*, which has been isolated from cow milk (Quigley et al., 2013) and has strains associated with (eponymously) steroid and broader aromatic hydrocarbon degrading (Kamimura et al., 2010; Horinouchi et al., 2018). *Ottowia beijingensis*, first isolated from phenol degrading consortia in wastewater sludge, and *Flavobacterium cucumis* have not previously been reported in breast milk, to the authors’ knowledge, but both have strains with hydrocarbon degradation capability (Al-Mailem et al., 2014; Cao et al., 2014; Fernandez-Gonzalez et al., 2018), consistent with the majority of differentially abundant species significantly enriched in late milk.

Other species significantly enriched in late milk have previously been isolated from an expected common source—the oral cavity, including *Prevotella melaninogenica*, *Veillonella dispar and Actinomyces odontolyticus*. *Actinomyces odontolyticus* is often considered pathogenic and strains have been associated with infection/mastitis (Hoyles et al., 2001; Bing et al., 2015), but it is also a common oral commensal species which has been observed to increase during puberty (Gusberti et al., 1990). *P. melaninogenica* is a common oral commensal bacteria which has been suggested as transmitted directly from mother to colonize the infant mouth via saliva (Könönen et al., 1994), and has also been suggested for the oral commensal *Granulicatella* (Dewhirst et al., 2010; Gomez and Nelson, 2017). Species such as *V. dispar*, identified here as significantly higher in relative abundance in late milk, has been recently discussed with relevance to infant age (being commonly isolated from the oral cavity) (Tuominen et al., 2018) and strains have been identified in late (3 months postpartum) milk alongside *Granulicatella* (Simpson et al., 2018) and uncharacterized species from the hard to culture TM7 group of bacteria Candidatus saccharibacteria (Cabrera-Rubio et al., 2012). These findings suggest important roles of poorly characterized and unknown bacteria associated to lactation stage, highlighting that a substantial amount is yet to be discovered about breast milk as an ecosystem.

### Limitations

Differentially abundant species are presented here based on amplified 16S rRNA gene sequences alone and do not establish viability of bacteria putatively identified. Future research confirming these findings should attempt to isolate species identified using culturing techniques. A concern often raised in milk microbiome studies relates to primer selection, an important limitation in bacterial marker technology. Inconsistency in the perceived “core” genera of bacteria commonly identified in the breast milk of healthy mothers is well captured in the systematic human breast milk microbiome reviews of Jeurink et al. (2012) and Fitzstevens et al. (2017) where most of the major studies report *Staphylococcus*, *Streptococcus, Pseudomonas* and either *Bifidobacterium* or *Cutibacterium* (formally *Propionibacterium*). This observed inconsistency between *Bifidobacterium* and *Cutibacterium* originates from the fundamental limitation of bacterial marker technology that there are no universal primers for the 16S rRNA gene (Klindworth et al., 2013; Gonzalez et al., 2019). Sequence analysis has revealed that the difference between these studies relates to a bias associated with primer selection. Specifically, the primers 27F/533R (utilized in this study), targets the V1-V3 region of the 16S rRNA gene and are often used in breast milk microbiome studies (Cabrera-Rubio et al., 2012; Mediano et al., 2017; Lackey et al., 2019) can successfully amplify the putative “core” breast milk genus *Cutibacterium* (formally *Propionibacterium*) (Hunt et al., 2011; Jost et al., 2013; Jiménez et al., 2015) but cannot amplify species from the putative “core” genus *Bifidobacterium* (Klindworth et al., 2013). Conversely, the other most commonly used primers, the 515F/806R primer pair (V4 region targeting), do amplify species within the genus *Bifidobacterium* but do not amplify species from the genera *Cutibacterium* (Klindworth et al., 2013), although *Bifidobacterium* sp. are often (Moossavi et al., 2019) but not always (Davé et al., 2016; Ramani et al., 2018) identified in breast milk studies when such primers are used. Whole genome shotgun (WGS) approaches can serve to resolve these types of identification bias generated from 16S rRNA primer selection; however, quantitative differential abundance analysis using WGS approaches remains a technical challenge for studies involving high sample numbers and with high bacterial diversity, such as is present within breast milk. Although *Pseudomonas* sp. are normally identified in breast milk of healthy mothers (Martín et al., 2007; Hunt et al., 2011, 2012; Jost et al., 2013; Ward et al., 2013; Jiménez et al., 2015; Urbaniak et al., 2016; McGuire and McGuire, 2017; Moossavi et al., 2019) species from the genera are common in the environment and could represent contamination (Jiménez et al., 2015; Treven et al., 2019), particularly as late breast milk samples had lower average DNA concentrations, although recent evidence suggests *Pseudomonas* presence in breast milk is genuine (Douglas et al., 2020).

## Conclusion

Evidence is provided of extensive remodeling of the breast milk microbiome across lactation stages in a cohort of healthy unrelated Guatemalan mothers. These include a microbiome shift from specific *Staphylococcus* and *Streptococcus* species at the beginning of lactation toward *Sphingobium* and *Pseudomonas* species at a later stage of lactation. Collectively, the findings reveal that common changes occur in the healthy human breast milk microbiome over the first 6 months of lactation and highlight the importance of investigating the microbiome at different stages of lactation. The large majority of species significantly associated with later lactation, such as *Sphingomonas yanoikuyae*, *Pseudomonas putida*, *Stenotrophomonas maltophilia*, *Cloacibacterium normanense*, *Comamonas testosteroni*, *Ottowia beijingensis*, and *Flavobacterium cucumis* are intriguing in having the common functional trait of aromatic hydrocarbon degradation. Whilst the contribution of specific bacterial species to infant metabolism and immunity is largely opaque, these distinct changes suggest active on-going remodeling of the milk microbiome throughout lactation that could contribute favorably to infant health. Moreover, these insights illustrate the need to study under-represented human communities in breast milk research given the complexity of cross-talk between the mother-infant dyad during lactation.

## Data Availability Statement

All data are made available within the manuscript or Supplementary Data. Raw sequence data has been deposited at the European Genome-Phenome Archive (EGAD00001004160) and are available upon request to KK, kristine.koski@mcgill.ca.

## Ethics Statement

The studies involving human participants were reviewed and approved by McGill Institutional Review Board and CeSSIAM Human Subjects Committee. All participating mothers provided written informed consent for participation.

## Author Contributions

EG and NB analyzed microbiome data, created figures, and drafted the manuscript. CL drafted the manuscript, prepared samples for the 16S rRNA sequencing and LL prepared data files of maternal characteristics. NS supervised milk sample collection. KK, CL, LA, and MS conceptualized the study design and edited the final manuscript. NS, LA, and KK provided the financial support. All authors contributed to the article and approved the submitted version.

## Conflict of Interest

The authors declare that the research was conducted in the absence of any commercial or financial relationships that could be construed as a potential conflict of interest.

## Supplementary Material

Supplementary File 1 Total amplified community flower diagram illustrating raw count distribution across all OTUs. – Count correlation of 200 OTUs including all mothers. – *Streptococcus mitis* strain genome clustering. *Streptococcus mitis* DA is strain cluster. Strain level research has been conducted (Jensen et al., 2016) but this tree is based on all the up-to-date genomes submitted to NCBI.

Supplementary File 2 OTU table and lactation DA table.
