# Supplementary material for: Distinct Changes Occur in the Human Breast Milk Microbiome Between Early and Established Lactation in Breastfeeding Guatemalan Mothers
Source: Front Microbiol. 2021 Feb 12;12:557180. doi: 10.3389/fmicb.2021.557180 (PMC7907006; doi:10.3389/fmicb.2021.557180)
Supplement: Supplementary file 2 [file Data_Sheet_1.pdf]

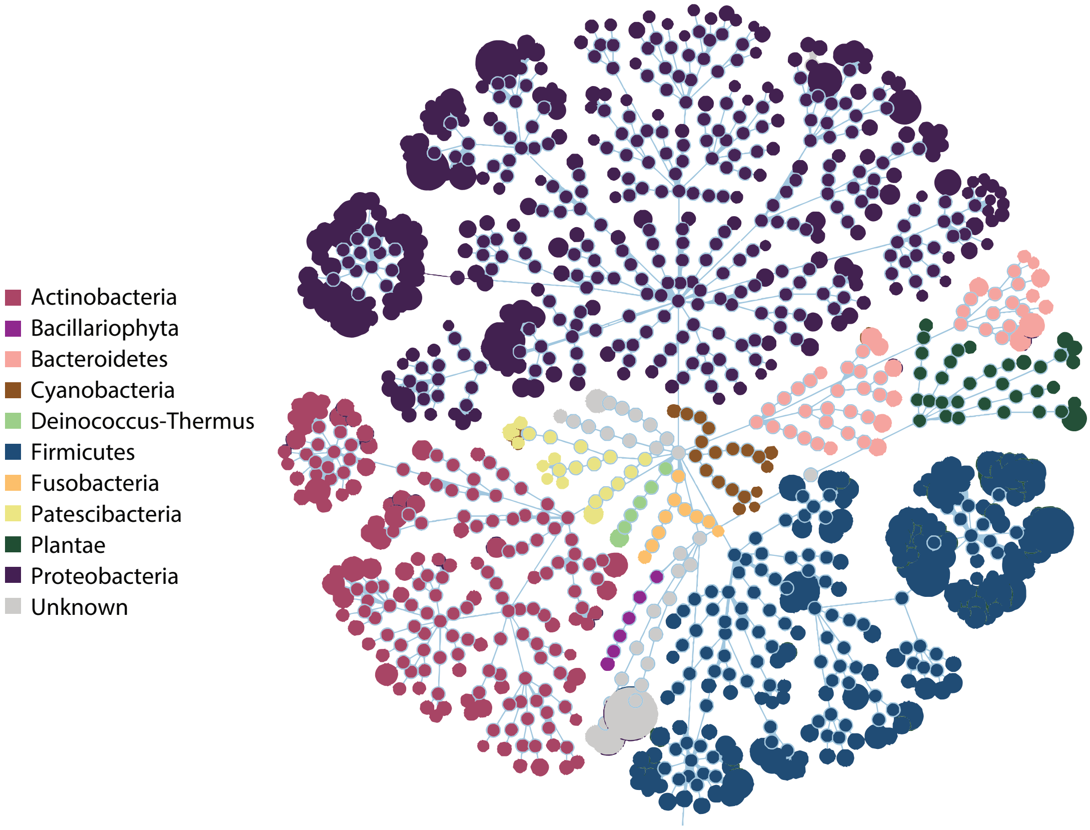

Supplementary figure 1 Total amplified community flower diagram illustrating raw count distribution across all OTUs.

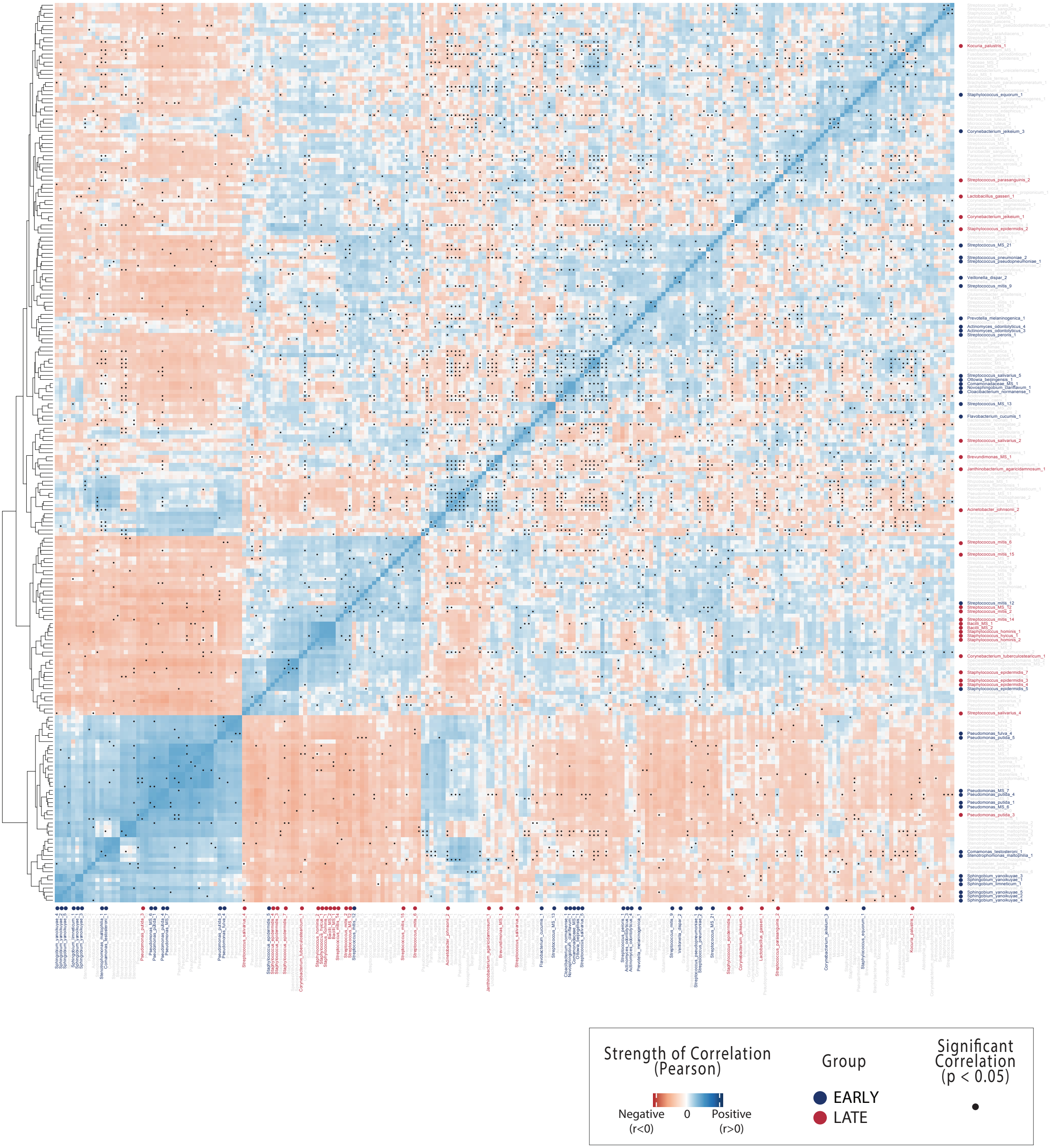

Supplementary figure 2 Count correlation of 200 OTUs including all mothers.

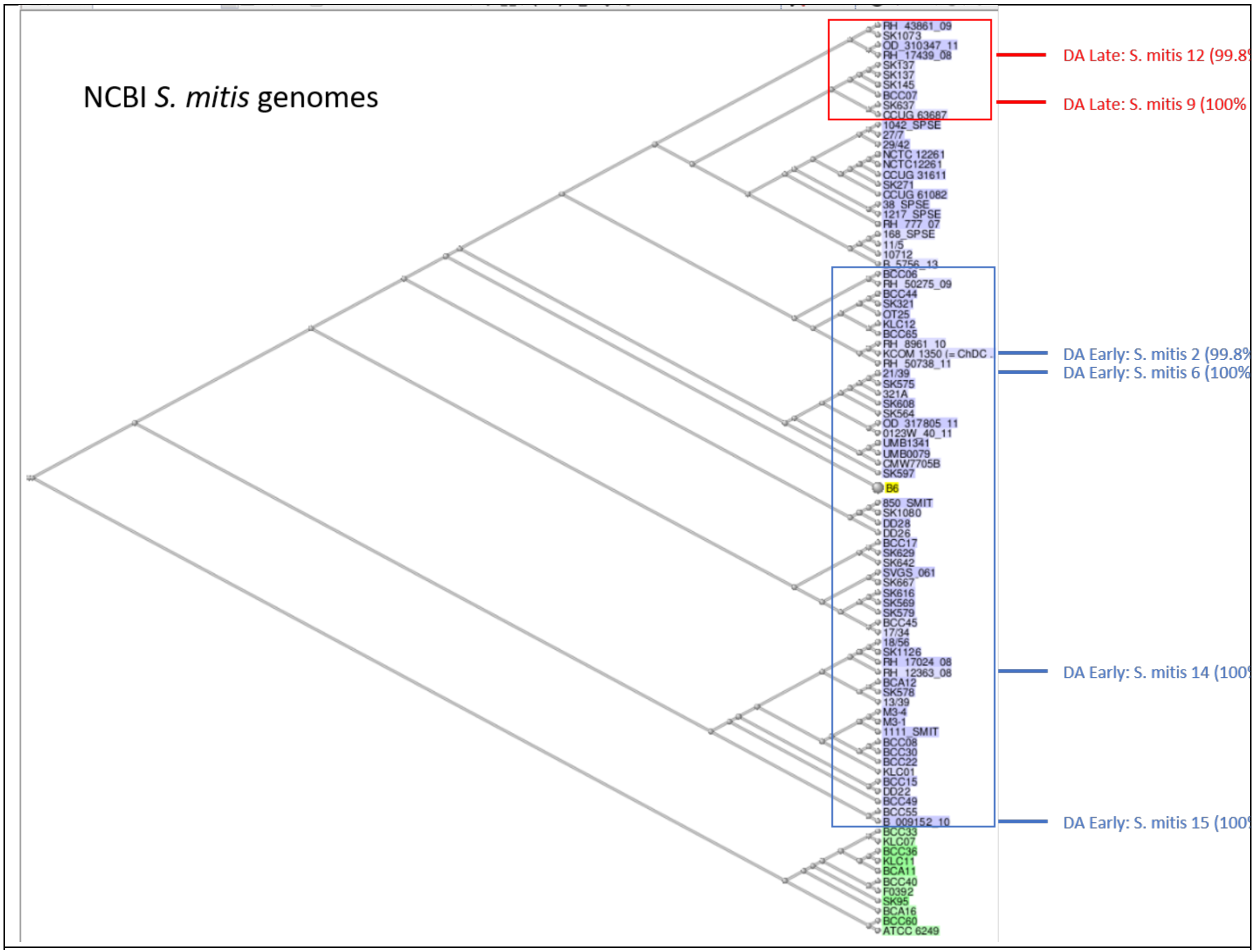

Supplementary figure 3 *Streptococcus mitis* strain genome clustering

Streptococcus mitis DA is strain cluster. Strain level research has been conducted (110) but this tree is based on all the up-to-date genomes submitted to NCBI.
